# Tight glycemic control in the ICU - is the earth flat?

**DOI:** 10.1186/cc13950

**Published:** 2014-06-27

**Authors:** Garry M Steil, Michael SD Agus

**Affiliations:** 1 Division of Medicine Critical Care, Boston Children’s Hospital and Harvard Medical School, Boston, MA 02115, USA

## Abstract

Tight glycemic control in the ICU has been shown to reduce mortality in some but not all prospective randomized control trials. Confounding the interpretation of these studies are differences in how the control was achieved and underlying incidence of hypoglycemia, which can be expected to be affected by the introduction of continuous glucose monitoring (CGM). In this issue of *Critical Care*, a consensus panel provides a list of the research priorities they believe are needed for CGM to become routine practice in the ICU. We reflect on these recommendations and consider the implications for using CGM today.

## 

*Continuous glucose control in the ICU: report of a 2013 Round Table meeting*, published in this issue of *Critical Care*[1], summarizes the discussion and recommendations of a round table meeting on the management of blood glucose levels in the ICU. The self-selected panel of authors recommends eight areas where it believes research is needed, beginning with head-to-head comparisons of different continuous glucose monitoring (CGM) devices and ending with randomized controlled studies validating closed-loop insulin delivery.

Appropriately, the recommendations focus on what is needed to advance CGM into the ICU and do not address whether tight glycemic control is beneficial or what the appropriate target range should be. Nonetheless, the answers to these questions will impact the importance of the recommendations. Of the prospective randomized controlled studies performed to date, many have failed to show a clinical benefit to tight glycemic control (TGC) - including our own study in children less than 3 years of age following cardiac surgery [2]. Our current study assessing the possible benefit of TGC in hyperglycemic critically ill children with cardiovascular and/or respiratory failure (NCT01565941) seeks to answer the question whether control in the target range 80 to 110 mg/dL results in better outcomes than control in the 150 to 180 mg/dL target range. Clearly, if the 80 to 110 mg/dL range proves beneficial, the need to introduce CGM into the ICU will be paramount as this target range is difficult to achieve without increasing the incidence of hypoglycemia. This may be less important if the 150 to 180 mg/dL range is shown to be equally effective. It is possible that TGC with CGM will reduce glucose variability irrespective of the target range, and the panel’s recommendations appropriately call for study of the effect of different treatment algorithms on this metric. However, it should be noted that the evidence the authors cite supporting the importance of glycemic variability [3] is based on retrospective analysis, which cannot be used to infer causality.

Still, the question remains as to how best to manage glucose levels in critically ill patients today. Putting aside whether control in a low target range is better than in a higher range, or whether a reduction in glucose variability *per se* improves clinical outcomes, there are low and high glucose levels that would be treated today in virtually every ICU. Every effort needs to be made to avoid these ends of the spectrum. To this end, one might ask whether CGM devices should be used in the ICU now. One can correctly infer from the recommendations that there have been no head-to-head comparisons of different CGM devices, and that the trends reported have also not been validated. Likewise, investigators who have established insulin protocols at their institutions might ask whether the protocols need to be re-evaluated given the marked differences in insulin recommendations noted by Wilson and colleagues [4] in work highlighted by the consensus panel. Our own review of TGC protocols concurs with that of Wilson and colleagues [4] in that we also noted substantive differences among the existing protocols [5]; however, we concluded that virtually all the protocols could be expected to achieve and maintain their desired target glucose ranges and each could be reasonably expected to benefit from the use of CGM devices.

## Conclusion

We emphasize again that the benefit of TGC in the ICU has not yet been established, and argue that equipoise for this and other questions related to TGC in the ICU be maintained. We agree more studies of CGM in the ICU are needed, but encourage investigators to consider whether the existing CGM devices might reduce the extreme high or low glucose values that occur during routine care. We conclude with a statement put forth by Dr Kavanagh [6] in his editorial accompanying our study of TGC in young children [2] in which he states: "Perhaps the most important question from a decade of studying glucose control in the ICU is how influential practice guidelines advocating tight glucose control were developed yet turned out to be harmful … guideline writers, reflecting on the experience, must accept that there are multiple sources of clinical knowledge. Clinicians in turn should use guidelines wisely, recognizing that no single source of knowledge is sufficient to guide clinical decisions." We agree and would point out there is an inherent danger in consensus opinion replacing clinical evidence. At one point in history the consensus was that the earth was flat.

## Abbreviations

CGM: continuous glucose monitoring; TGC: tight glycemic control.

## Competing interests

In the past 5 years MSDA has consulted to Roche Diagnostics and Medtronic Diabetes on glucose measurement technologies for use in the hospital setting, and Dexcom and Nova Biomedical have contributed devices or discounted devices for use in investigator-initiated randomized controlled trials conducted by MSDA and GMS.
